# The Importance and Challenges of Early Diagnosis of Paraneoplastic Skin Syndromes in Cancer Detection—A Review

**DOI:** 10.3390/cancers17071053

**Published:** 2025-03-21

**Authors:** Aleksandra Rościszewska, Kamila Tokarska, Aleksandra Kośny, Paulina Karp, Wiktoria Leja, Agnieszka Żebrowska

**Affiliations:** Department of Dermatology and Venereology, Medical University of Lodz, Hallera 1, 90-647 Lodz, Poland; aleksandra.rosciszewska@umed.lodz.pl (A.R.); kamka1990@o2.pl (K.T.); asiudak145@gmail.com (A.K.); paulina.karp@student.umed.lodz.pl (P.K.); wiki.leja60@gmail.com (W.L.)

**Keywords:** cancer, cutaneous paraneoplastic syndromes, skin disorders, differentiation

## Abstract

Skin paraneoplastic syndromes (SPNSs) are a group of disorders that arise as a consequence of cancer but are not directly related to the tumor mass itself. This review aims to provide a comprehensive overview of these syndromes, encompassing their pathophysiology, clinical features, diagnostic approaches, differential diagnosis, and management strategies. These syndromes, which include conditions such as Bazex syndrome, acanthosis nigricans, dermatomyositis, and necrolytic migratory erythema often manifest prior to or concurrently with a cancer diagnosis, serving as potential early warning signs of underlying malignancies. This review delves into the spectrum of SPNSs and their associations with specific cancer types. Special emphasis is placed on the critical role of dermatologists and oncologists in identifying these skin manifestations as potential markers of malignancy. By raising awareness of SPNSs, this paper highlights the pivotal importance of prompt recognition and intervention in reducing cancer-related mortality.

## Simple Summary

Skin paraneoplastic syndromes (SPNSs) are skin conditions linked to cancer but not caused by the tumor itself. These syndromes, such as erythroderma, pyoderma gangrenosum or acanthosis nigricans, often appear before or alongside cancer, serving as early warning signs. Early detection is crucial for prompt cancer treatment. However, diagnosing SPNSs is challenging due to their rarity and diverse symptoms. This article stresses the importance of healthcare professionals, particularly dermatologists and oncologists, in recognizing these signs. This article also highlights diagnostic criteria like Curth’s criteria to connect skin conditions with underlying malignancies and improve early cancer diagnosis and patient outcomes.

## 1. Introduction

Paraneoplastic skin syndromes (PNS) represent a fascinating and critical intersection between dermatology and oncology. These rare dermatological manifestations can be the first sign of an underlying malignancy, offering a unique opportunity for early cancer detection and intervention. Recognizing PNS is paramount, as these cutaneous indicators may precede the diagnosis of the primary tumor by months or even years, potentially altering the clinical course and prognosis of patients. However, the diagnostic process is fraught with challenges. The rarity and diverse presentation of PNS often lead to misdiagnosis or delayed recognition, complicating the clinical picture and delaying crucial oncological evaluations.

**Figure 1:**
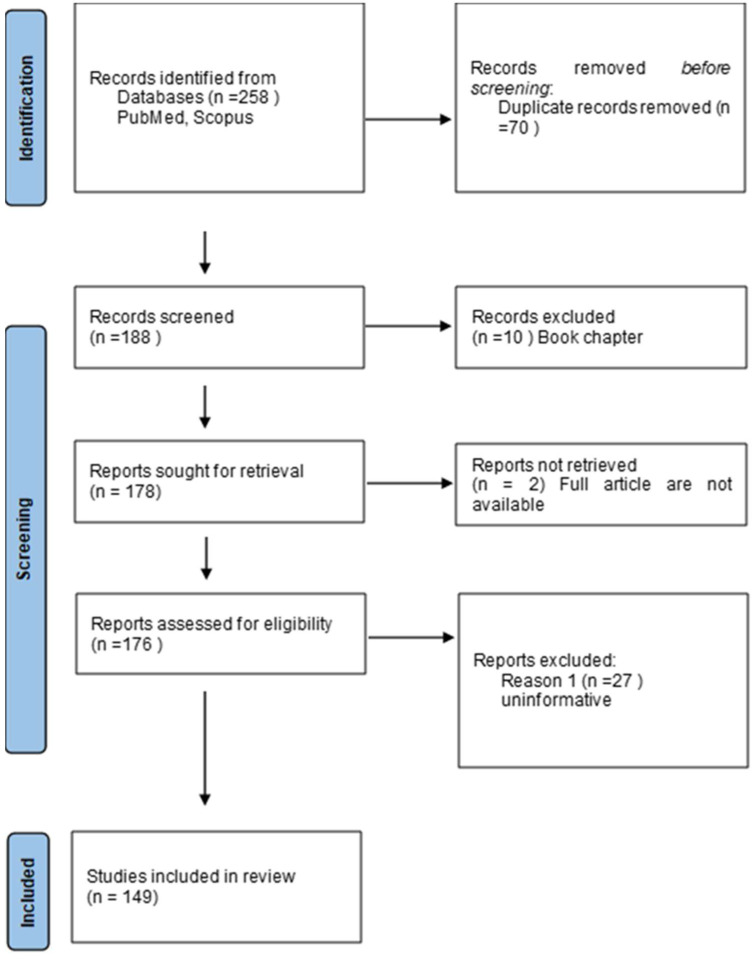
Flowchart on the selection and evaluation of scientific articles.

This article explores the significance of early identification of paraneoplastic skin syndromes in the context of cancer diagnosis and highlights the challenges that healthcare providers face in recognizing these elusive conditions. Numerous skin lesions may be associated with tumors, often malignant. These lesions can present as direct metastases; however, there are also several syndromes that are not composed of the same cells as the primary tumor. Despite this, their occurrence is closely linked to specific types of malignancies. Our review focused on a selection of the most common syndromes, which often present a diagnostic challenge in physician practice. We aim to make reader think about paraneoplastic syndromes when the diagnosis is difficult, the course of the disease is atypical or treatment is problematic. To discuss the most common cutaneous paraneoplastic syndromes, we conducted a thorough search of the PubMed and Scopus databases using keywords for each syndrome. We selected articles based on their quality and date of publication between 1964 and 2025, so that this article provides a broad overview of current knowledge. A research study on PNS was conducted on 21 January 2023 by A.K., P.K., W.L., K.T. and A.R. The search included the terms “paraneoplastic pemphigus, “erythema gyratum repens”, “necrolytic migratory erythema”, “Bazex syndrome”, “acute febrile neutrophilic dermatosis”, “pyoderma gangrenosum”, “exfoliative dermatitis”, “dermatomyositis”, “Leser–Trélat syndrome”, “acanthosis nigricans”, “paraneoplastic skin syndromes” and “treatment” in the articles written in English or Polish in the PubMed database; in total, 258 potentially eligible articles were detected. Additional relevant publications were obtained by reviewing the references from the chosen articles. After the removal of duplicates and irrelevant articles based on the titles, abstracts, and book chapters, 149 original articles were included in this review (Figure 1).

There are some criteria which are helpful in making the diagnosis of paraneoplastic syndromes, which is Curth’s criteria [1,2]. At least one of them must be met to associate cutaneous lesion with underlying malignancy:

1. Coexistence of dermatoses and cancer.
2. Simultaneous development/resolution with the primary tumor. The reappearance of skin lesions indicates advancement of the cancer.
3. There is a distinct relationship between the type of tumor and the type of skin eruption. A particular malignancy is consistently linked with a specific skin condition.
4. Reliable case–control studies demonstrate a significant statistical link between the type of cancer and the skin condition.
5. There is a genetic link between skin disorders and cancer.

Among paraneoplastic syndromes, we can distinguish those that do not pose diagnostic difficulties, but also those that may imitate other skin diseases in their appearance. In this situation, histopathological examination may be conclusive. It is necessary to know that skin changes may by connected with internal malignancy, because this may lead to cancer being diagnosed sooner and starting treatment at an early stage which may provide a greater chance of recovery.

Paraneoplastic syndromes which can be problematic in recognition include erythema gyratum repens, necrolytic migratory erythema, Bazex syndrome, Sweet syndrome, pyoderma gangrenosum and erythroderma. There are also some syndromes which lead to simple diagnosis and always require cancer screening, such as paraneoplastic pemphigus, dermatomyositis, Leser–Trélat syndrome or acanthosis nigricans.

## 2. Paraneoplastic Syndromes That Pose Diagnostic Challenges

Erythema gyratum repens (EGR) is a rare paraneoplastic syndrome which may cause diagnostic challenges. Erythema gyratum repens is strongly associated with internal malignancy but there are also rare reports of this condition existing without tumors. [3] Clinically, it is a rapidly (usually 1 cm a day) spreading superficial erythema that creates an unusual pattern of intersecting coils and spirals. Erythema gyratum repens resembles the rings of a cut tree, and it is sometimes referred to as “zebra skin” [4].

Typical localization of skin lesions are trunk and proximal extremities. The hands, feet and face are spared. Desquamation may be observed at the edge of the erythema. Desquamation spreads centrifugally. Most patients experience some degree of pruritus.

Histopathological examination is nonspecific. Histopathological examination may show mild to moderate hyperkeratosis, parakeratosis and spongiosis. The dermal vessels surrounded by lymphohistiocytic infiltrate with occasional eosinophils have been described. Mast cells may also be seen [5]. In some cases on direct immunofluorescence test (DIF), granular IgG and C3 deposition can be seen along the dermal–epidermal junction [6,7].

While the precise etiology of EGR remains unclear, several immunological mechanisms have been proposed to explain its pathogenesis. One hypothesis suggests that the tumor may trigger the production of antibodies that cross-react with the skin’s basement membrane. Alternatively, it has been proposed that the tumor could secrete polypeptides that bind to skin antigens, thereby making them immunogenic. Another theory involves the deposition of tumor antigen–antibody complexes on the basement membrane, potentially leading to a reactive dermatitis. The presence of immunofluorescence patterns involving IgG, C3, and C4 at the basement membrane supports the likelihood of an underlying immunological mechanism [3,8,9].

In nearly 82% of cases of EGR coexistence of internal malignancy have been reported. The most common are carcinoma of the lung, breasts, esophagus, cervix, stomach and pharynx. There are also some reports of prostate, bladder, intestinal, uterus and pancreas cancer. But some authors also described patients with EGR without underlying malignancy. In such cases, EGR was associated with bullous pemphigoid, CREST syndrome, tuberculosis and also pityriasis rubra pilaris [10,11]

The diagnosis of EGR is made mainly on the basis of the clinical appearance of the skin lesions. That is way it may provide to diagnostic difficulties. Sometimes the clinical pictures may resemble tinea corporis [12]; in these cases, we should perform a mycological examination of the lesion to exclude a fungal infection. Also a medical interview may be helpful if we are dealing with a farmer or an animal owner, as mycosis is more likely. Some skin lesions may resemble psoriasis, so a positive family history of psoriasis may be decisive. Subacute cutaneous/discoid lupus erythematosus, bullous pemphigoid, erythema migrans and erythema annulare centrifugum should also be considered in the differential diagnosis [13]. There is nonspecific treatment for EGR. Since the course of the disease parallels that of the underlying disorder, therapy should be targeted at the tumor.

Various dermatologic and immunosuppressive therapies have been employed in the management of EGR, but their effectiveness remains limited. Systemic corticosteroids are often ineffective, and topical steroids, vitamin A, and azathioprine have also failed to alleviate the cutaneous symptoms. Improvement or resolution of EGR, along with its associated severe pruritus, largely depends on the identification and treatment of the underlying malignancy. In cases of widely metastatic disease, the response of EGR to chemotherapy is variable. In some patients, the rash may only resolve in the terminal stage of the disease, typically coinciding with profound immunosuppression [3,14].

Necrolytic migratory erythema (NME) is also a rare paraneoplastic syndrome. This disease most commonly coexists with cancers, with the highest number of cases described as prevalent in glucagonoma. However, a single case has been reported in the literature of necrolytic migratory erythema coexisting in a patient with lung adenocarcinoma treated with erlotinib [15]. There are also cases described of coexistence with zinc deficiency, liver disease, or pancreatitis, as well as two cases of NME induced in patients with non-small-cell lung cancer treated with gefitinib [16,17].

In the pathogenesis of NME, amino acid deficiencies and deficiencies of other elements such as zinc are mentioned, which are directly related to elevated levels of glucagon in the blood. Since more than 90% of NME cases are associated with coexisting glucagonoma, patients often experience weight loss, diarrhea and general malaise. Typical skin lesions present as reddish-brown plaques that undergo superficial necrosis and crusting. Blistering reactions within the erythematous lesions and desquamation are often observed. The lesions can spread peripherally and typically affect the perioral region, trunk, extremities, and perineum. Nail and oral involvement are other associated manifestations. Patients often experience pruritus, pain, and a burning sensation at the site of the lesions. NME is one of the symptoms included in glucagonoma syndrome, in addition to glossitis, diabetes mellitus, angular cheilitis, venous thrombosis, weight loss, normochromic and normocytic anemia and neuropsychiatric symptoms. To help remember these classic characteristics, the acronym “4D” has been suggested, standing for dermatosis, diabetes, deep vein thrombosis, and depression [18,19]. In histopathological examination, some characteristic features for NME can be found. It is crucial to note that the histopathologic changes are confined entirely to the epidermis, especially the superficial epidermis. The hallmark of NME is the necrosis of the upper spinous layer of the epidermis. However, superficial epidermal necrolysis is relatively nonspecific and may only be focally present, most likely at the edge of an active lesion. Other significant findings in NME include irregular acanthosis, and loss of the granular layer. Confluent parakeratosis overlying vacuolated superficial keratinocytes is quite characteristic of NME. Additionally, there may be mild perivascular lymphocytic or neutrophilic infiltrate and intraepidermal bullae. Multiple biopsies should be taken if NME is suspected [20,21].

Dermatologic changes in NME may be misdiagnosed as they can suggest other dermatoses, such as acrodermatitis enteropatica. In this case, erythema and erosions are limited to areas around body orifices, but are responsive to zinc supplementation. Some cases of NME require differentiation from erythema multiforme (EM). However, EM is most often triggered by a specific factor (such as medications or herpes infection) and typically presents with a characteristic target or “bullseye” appearance.

These two conditions can also be distinguished by the good response of EM to removing the trigger and to systemic steroid therapy. Skin lesions in contact or atopic dermatitis can mimic those seen in NME. However, atopic dermatitis is often associated with a personal or family history of allergies, or there may be a specific trigger causing contact-related changes, like in contact dermatitis.

In the differential diagnosis, conditions such as erythrokeratoderma and psoriasis should also be considered. Also because of the ulceration in the groin area, Hailey–Hailey disease (HHD) must be taken into consideration during differentiation. To rule out HHD, a biopsy for histopathological examination should be taken. A family history of similar skin lesions can also be helpful in the differential diagnosis [22].

Improvement in skin lesion is achieved with surgical resection of coexisting tumor, chemotherapy and long-acting somatostatin [23].

Medical therapy plays a pivotal role in managing patients with metastatic disease at presentation or those who are not suitable candidates for surgical intervention. Cryoablation is a novel treatment modality aimed at stabilizing neuroendocrine tumors and consequently improving necrotizing erythema migrans. Cryoablation has been confirmed to be a potentially safer and more effective therapeutic option in the treatment of pancreatic tumors compared to classical pancreatic resections [24]. Palliative treatment options include long-acting somatostatin analogs (e.g., octreotide and lanreotide) or interferon-alpha. Somatostatin analogs, particularly, have demonstrated significant efficacy in case reports by antagonizing glucagon activity, effectively mitigating both necrolytic migratory erythema (NME) and systemic symptoms associated with glucagonoma. Lanreotide, administered as a monthly depot injection, has been shown to prolong progression-free survival in patients with metastatic gastrointestinal neuroendocrine tumors.

While somatostatin analogs provide symptom relief and enhance survival, therapy must be maintained indefinitely, as discontinuation often leads to the rapid recurrence of NME and other glucagonoma-related symptoms [25,26]. Chemotherapy with agents like streptozotocin and 5-fluorouracil can be used for palliation in metastatic disease, though it is generally less preferred due to the tumor’s poor responsiveness to current chemotherapeutic regimens. Biologic agents, including sunitinib and everolimus, have shown promise in clinical trials for treating pancreatic neuroendocrine tumors (pNETs), including glucagonomas, and may be appropriate in specific clinical scenarios [27].

Additionally, innovative treatments for liver metastases, such as liver resection, transplantation, percutaneous ablation, targeted radiotherapy, and chemoembolization, may also be considered to address metastatic disease in select cases.

Bazex syndrome can mean either acrokeratosis or a set of symptoms including the genetic syndrome of basal cell carcinoma, hypotrichosis, disorders of sweating and follicular atrophoderma. Acrokeratosis paraneoplastica (APB) was first described in 1965 by Bazex, as a condition accompanying cancer of the pyriform fossa [28].

**Figure 2:**
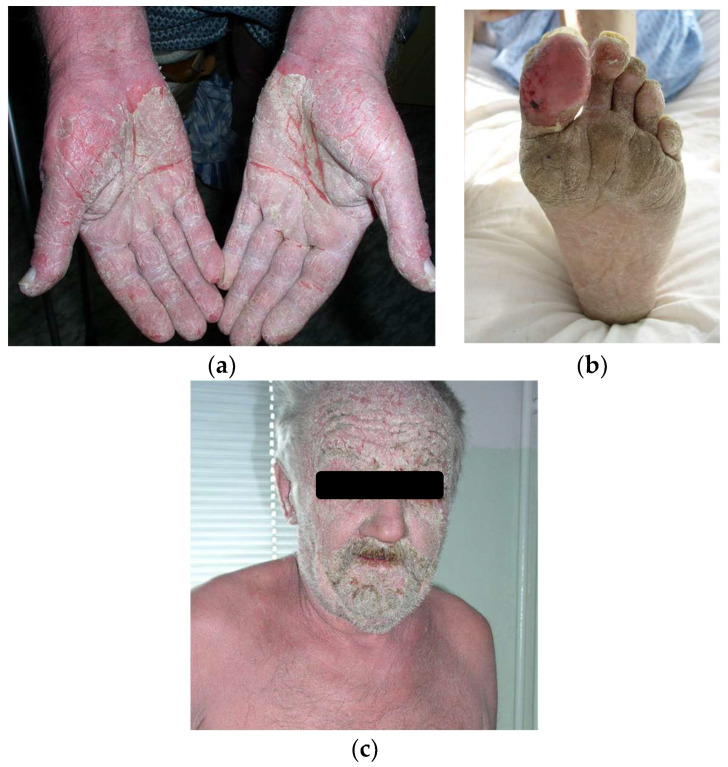
Skin lesions in Bazex syndrome. (**a**,**b**) The most commonly affected areas are the hands and feet. The lesions are erythematous with scaling and sometimes crack. (**c**) As the disease progresses, the lesions spread to other areas of the skin and may involve the facial skin.

In Bazex syndrome, the most commonly affected areas for skin lesions are the hands, feet, ears, and nose. These skin eruptions are characterized by scaly, hyperkeratotic, and red to purple lesions. According to Bazex, the disorder progresses through three stages. The first stage involves a symmetrical erythemato-squamous eruption on the fingertips and toes, as shown in Figure 2a,b. The changes may also affect the nose and ears and, as it progresses, the entire face (Figure 2c). The fingernails and toenails become abnormal, showing paronychia, subungual keratotic debris, and possibly onycholysis.

In the second stage, skin lesions spread inward. Areas such as the forehead, cheeks, knees, elbows and thighs may be affected, with the borders of the skin lesions being blurred. The lesions may be accompanied by itching, usually of mild intensity, which stimulates scratching. This can lead to slight bleeding. In the third stage, the lesions may already be noticeable on the limbs and torso. Changes resembling psoriasis are often described, with a tendency to develop fissures. In the early phases of the disease, metastasis to the cervical or mediastinal lymph nodes can already be observed, but the primary tumor is usually asymptomatic. In the later stages, both the underlying tumor and the affected lymph nodes are usually noticeable [29,30,31].

The exact pathogenesis of Bazex syndrome remains unclear. A suggestion has been made regarding a shared immune response to an antigen between the tumor and the skin. One hypothesis proposes that coexisting malignancies may induce an immune response shifted towards a Th-2 response, which in turn could lead to increased expression of epidermal growth factor receptors (EGFR) in the affected keratinocytes. An immunological basis is supported by some researchers through the detection of immunoglobulins (IgG, IgM, IgA) and complement components (especially C3) along the basement membrane of both the skin affected by lesions and the skin free from changes [32].

Histological features in Bazex syndrome are unspecific. Parakeratosis, hyperkeratosis, isolated necrosis of keratinocytes, acanthosis, and a perivascular lymphohistiocytic inflammatory infiltrate may occur. The immunofluorescence studies of lesional skin from patients with acrokeratosis paraneoplastica usually give negative results [33].

In the review written by Shah and Ferazzano (2023), the cancers in which the coexistence of APB has been described so far were summarized. Among them, the most common were: lung cancer, oral cancer, hand and neck cancer, esophageal carcinoma and just a few cases of hepatocellular, lip, pancreatic, cervical, gastric, colorectal and breast cancer, but also follicular lymphoma, T-cell lymphoma and acute myeloid leukemia [34]. The strong connection between APB and internal malignances, makes APB an essential indicator of hidden cancers.

Early detection is crucial for timely and effective treatment. The differential diagnosis primarily includes psoriasis. In this case, it is important to consider the recurrent nature of the lesions, the triggering factor preceding the appearance of skin changes (e.g., stress, infection, and alcohol abuse), or family history, which may indicate a diagnosis of psoriasis. It can also be helpful to take skin scrapings for mycological examination or to perform PAS staining in a histopathological examination to rule out fungal infection. Hyperkeratotic skin lesions on the soles of the feet may also suggest a diagnosis of eczema. In such cases, patch tests can be useful to exclude potential allergic causes. A histopathological examination should be decisive in that case. There have been case reports of acrokeratosis paraneoplastica-like findings in patients with systemic lupus erythematosus [35].

APB is characterized by resistance to treatment. Medications that have been tried and did not work include topical keratolytics, corticosteroids, tar, antifungals, and antibiotics.

Some successful treatments have been reported, such as oral etretinate and oral prednisone, but typically, removing the underlying cancer is necessary to clear the skin [36].

The preferred approach to resolving the described skin lesions is effective treatment of the underlying tumor. Paraneoplastic skin lesions, such as those observed in Bazex syndrome, typically do not respond to standard dermatological therapies for inflammatory skin diseases. However, there have been reports of improvement with treatments like vitamin D3, salicylic acid, and topical or systemic corticosteroids. Reported topical treatments include corticosteroids (e.g., clobetasol 0.05% or betamethasone 0.01%), salicylic acid 10% in Vaseline, itraconazole, isosorbide dinitrate, fluconazole, cephalexin, keratolytic agents, neomycin, nystatin, zinc ointment, antibiotics, and emollients. Additionally, some authors have suggested the use of oral dexamethasone (10 mg/day), which has shown favorable outcomes in managing the cutaneous lesions.

In recent years, anecdotal evidence has highlighted the potential benefits of systemic and topical retinoids for these skin lesions. Early administration of acitretin has been reported to effectively improve acrokeratosis paraneoplastica in patients with incurable primary malignancies [37]. Retinoids may also be used in combination with oral corticosteroids. Furthermore, some studies have suggested that psoralen combined with ultraviolet light (PUVA) therapy could be beneficial for selected patients with localized skin lesions.

There are also reports indicating that zinc supplementation might help alleviate these skin manifestations.

Acute febrile neutrophilic dermatosis (Sweet syndrome)was for the first time described by Dr. Robert Douglas Sweet in 1964 [38]. Neutrophilic dermatoses are a diverse group of inflammatory skin conditions, encompassing Sweet syndrome (SS), pyoderma gangrenosum, and subcorneal pustular dermatosis. Among these, Sweet syndrome is the most common [39].

We can distinguish three clinical types of the disease, which is classic (or idiopathic) SS, drug-induced SS and malignancy-associated type. Typical eruptions consist of red, painful, well-demarcated plaques or nodules. The lesions are typically referred to as pseudovesicular or pseudopustular, though they can also present as fully pustular, bullous, or ulcerative. They may develop on the face, neck, back, chest and also extremities. Mucosal changes are not specific for Sweet syndrome and occur in 3–30% of cases. In addition to skin lesions, the following may occur but rarely: arthritis, eye, kidney, CNS, lung, bones and liver involvement [40]. Also pyrexia and general malaise may be present.

Distinctive criteria first described by Su and Liu [41] and modified by von den Driesch are helpful in making the diagnosis of Sweet syndrome.

Two major criteria are identified: the rapid onset of tender, erythematous nodules or plaques, and the presence of neutrophilic infiltrates in histopathological examination that do not fulfill the criteria for leukocytoclastic vasculitis.

Minor criteria include fever over 38 degree; association with hematologic or visceral cancers, inflammatory conditions, or pregnancy, or occurring after an upper respiratory tract infection, gastrointestinal infection, or vaccination; rapid response to treatment with systemic corticosteroids or potassium iodide; deviations in laboratory tests (three of four): increased C-reactive protein level, level of leukocytes over 8000, with over 70% neutrophils; a erythrocyte sedimentation rate >20 mm/h.

To diagnose classical SS, both major and two minor criteria must be met.

In cases associated with malignancy, the condition should either precede, follow, or occur simultaneously with the diagnosis of the patient’s neoplasm, while also fulfilling classical Sweet syndrome diagnostic criteria [42].

Histological examination from the lesions should be helpful to make diagnosis. Distinguishing findings are: the presence of a widespread neutrophilic infiltrate in the dermis, fragmentation and edema of neutrophil nuclei. The main cells constituting the infiltrate in the dermis of skin lesions in SS are mature neutrophils. Occasionally, eosinophils, lymphocytes or histiocytes may also be found in the inflammatory infiltrate. Neutrophils can also appear in the epidermis as neutrophilic spongiotic vesicles or subcorneal pustules; however, if the infiltrate extend into the subcutaneous tissue and hypodermis, affecting adipocyte, it has recently been linked to myeloid disorders and it is called “subcutaneous Sweet syndrome” [43].

Many cases of coexistence of Sweet syndrome with cancer have been described in the literature, for the first time represented by Shapiro et al. [44]. The most common are leukemia, particularly acute myeloid or myelomonocytic leukemia [45]. SS may also been associated with chronic myeloid leukemia [46]. From the solid tumor. the most frequently found is embryonal carcinoma of testis, ovarian carcinoma, gastric carcinoma and adenocarcinoma of breast, prostate, and rectum [47]. Unlike most other paraneoplastic diseases, this syndrome may occur at an early stage of development of these cancers that cure is possible. Even though there are specific criteria to help identify Sweet syndrome, it still poses diagnostic difficulties.

There is also a well-known association between Sweet syndrome and infectious diseases, particularly upper respiratory syndrome and gastrointestinal infections. Cases of simultaneous tuberculosis, HIV infection, and hepatotropic viral infections have also been described. In addition, Sweet syndrome may be linked to autoimmune conditions, including rheumatoid arthritis, Behçet’s disease [48], inflammatory bowel diseases, and systemic lupus erythematosus.

Drug-induced Sweet syndrome is a well-recognized condition, with numerous medications implicated in its onset. Granulocyte colony-stimulating factor is the most commonly associated drug, but others include antibiotics (e.g., minocycline, nitrofurantoin, trimethoprim-sulfamethoxazole, norfloxacin, and ofloxacin), antihypertensives (e.g., hydralazine and furosemide), nonsteroidal anti-inflammatory drugs (e.g., diclofenac, celecoxib), immunosuppressants (e.g., azathioprine), [49] antiepileptics (e.g., carbamazepine and diazepam), anticancer agents (e.g., bortezomib, imatinib mesylate, ipilimumab, lenalidomide, topotecan, and vemurafenib), antipsychotics (e.g., clozapine), and antithyroid drugs (e.g., propylthiouracil). In these cases, Sweet syndrome typically develops after exposure or re-exposure to the offending drug and often resolves after drug withdrawal, with or without corticosteroid therapy.

Pregnancy is associated with the development of Sweet syndrome in approximately 2% of cases. The prognosis in pregnancy-related cases is favorable, with spontaneous resolution occurring after delivery and no reported maternal or infant morbidity or mortality [50].

The most problematic in differentiation is erythema nodosum (EN), especially when skin lesions are located in lower extremities. In both cases, we are dealing with painful, erythematous nodules, where histopathological examination is not always crucial. Accompanying symptoms in EN may also be similar (i.e., fever) or increased inflammatory markers [42]. Another disease which can be problematic in differentiation is toxic pustuloderma, which is a serious hypersensitivity reaction to medications like carbamazepine. Symptoms may include fever, an elevated white blood cell count, and involvement of the lymph nodes, liver, and kidneys. The histological appearance can resemble pustular SS; however, the presence of widespread, primarily isolated pustules, with or without association with hair follicles, helps to distinguish between the two conditions. When distinguishing it from SS, it is important to also consider: periarteritis nodosa, granuloma faciale, leukocytoclastic vasculitis, erythema elevatum diutinum, and infectious disease such as erysipelas or impetigo contagiosum. All of this disorders can have similar histopathological features, but rather vary in another symptoms.

Paraneoplastic SS may develop more problems with diagnosis, because general malaise and pyrexia are not obligatory in this cases. In some patients with classical Sweet syndrome, lesions can persist for weeks to months without treatment but eventually heal on their own. While there are no established guidelines for treating Sweet syndrome, systemic corticosteroids are usually the first choice for treatment. For patients who cannot use corticosteroids, oral therapy with potassium iodide or colchicine often leads to a quick resolution of Sweet syndrome symptoms and lesions [51]. In cases of malignancy-associated Sweet syndrome, the skin condition sometimes resolves after the related cancer goes into remission.

The differential diagnosis should include psoriasis vulgaris, psoriasis guttata, and viral exanthema [52].

The primary objective of pharmacotherapy in acute febrile neutrophilic dermatosis is to minimize morbidity and prevent complications. Systemic corticosteroids are considered the most effective first-line treatment, with topical corticosteroids being appropriate for localized lesions. In cases where corticosteroids are contraindicated, alternative options include anti-inflammatory or immunosuppressive agents. Potassium iodide and colchicine are also given as alternative first-line therapies [53].

Pyoderma gangrenosum (PG)—is a rare, autoinflammatory neutrophilic dermatosis, that occurs with equal frequency in both sexes. This disease typically co-occurs with other autoimmune disorders, with the most common being arthritis, inflammatory bowel disease, and paraproteinemia. Pyoderma gangrenosum (PG) can also present as a paraneoplastic syndrome, most frequently associated with myeloproliferative neoplasms (acute myeloid leukemia, acute/chronic lymphatic leukemia, myelodysplastic syndrome, T-cell lymphoma), and less commonly with solid tumors such as breast, colon, bladder, or prostate cancer [9].

**Figure 3:**
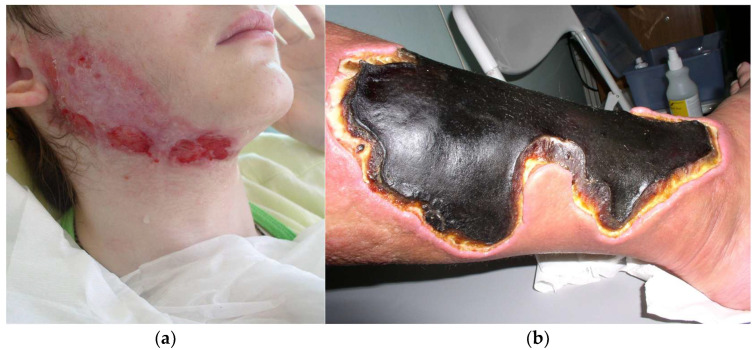
Pyoderma gangrenosum. (**a**) Ulcerations and erythema in the surrounding area located on the face. (**b**) Ulceration covered with necrotic tissue located on the tibial area.

Pyoderma gangrenosum comes in four main clinical types: ulcerative, bullous, pustular and vegetative (or superficial granulomatous). Skin lesions typically start as one or more red pustules, nodules or blisters that can rapidly develop into painful, irregular ulcers with undermined edges. Ulceration is often covered with necrotic tissue. “Sieve sign” is also a typical feature. The surrounding is covered with erythema [54]. In the case of PG, a pathognomonic sign known as pathergy is observed, meaning that new lesions can develop as a result of minor injuries [55]. Ulcers are located mainly on the lower limbs and trunk, but they can also develop in any part of the body. Figure 3a,b present skin lesions associated with PG in typical locations.

The findings in histopathological examination depending on the location and stage of the lesions. Biopsy samples from the ulcer’s edge typically reveal neutrophils and perivascular lymphocytic infiltrates along with dermal edema, while samples taken from the center of the ulcer predominantly show a neutrophilic infiltrate. It is common to observe vascular damage characterized by fibrin deposition, thrombosis, and red blood cell extravasation [56].

PG is typically diagnosed by ruling out other conditions, as there are no clear-cut laboratory tests or histopathological markers for it, which often results in it being misdiagnosed [57].

The criteria established following a Delphi consensus exercise in 2018 replaced the Su criteria, introducing a single major criterion for diagnosing pyoderma gangrenosum: the presence of a neutrophilic infiltrate. When combined with four or more of the eight minor criteria, this approach achieves a sensitivity of 86% and a specificity of 90% for the condition [58].

Major criterion

1. Histopathology of ulcer edge must show a neutrophilic infiltrate.

Minor criteria

1. Exclusion of infection;
2. Pathergy;
3. History of inflammatory bowel disease or inflammatory arthritis;
4. History of papule, vesicle, or pustule ulcerating within four days;
5. Peripheral erythema, undermining border, and tenderness at the ulcer site;
6. Multiple ulcers, at least one on the anterior lower leg;
7. Cribriform or wrinkled paper scars at the site of the healed ulcer;
8. Decreased size of the ulcer within one month of initiating immunosuppressive medication.

In the differential diagnosis, it is essential to consider other causes of chronic ulcers, such as venous or arterial insufficiency, Sweet syndrome, antiphospholipid syndrome, systemic lupus erythematosus, venous or arterial ulcers, tuberculosis, sporotrichosis, ulcerating skin tumors and lymphomas or vasculitis [57]. There are some features which can lead to PG suggestion, such as repeated negative cultures results, failure to respond to antibiotics and neutrophilic infiltration in skin biopsy [59].

In the treatment of the acute, initial phase of PG, systemic steroids are used. For chronic therapy, immunosuppressive drugs are chosen, most commonly cyclosporine A, dapsone, colchicine, and TNF-alpha inhibitors. It is important to perform surgical wound debridement carefully, as the pathergy phenomenon can cause the formation of new ulcers. There are also reported cases of spontaneous healing of PG-related ulcers following the successful treatment of an associated malignancy [60].

Exfoliative dermatitis (erythroderma) is an inflammatory skin condition that affects more than 90% of the skin’s surface. Exfoliative dermatitis is not a disease in the strict sense, but a symptom that can accompany other medical conditions. Erythroderma may result from the exacerbation of pre-existing dermatological conditions such as psoriasis, atopic dermatitis, pityriasis rubra pilaris and cutaneous lymphoma [61]. However, it can also be a symptom of a drug reaction or occur as a paraneoplastic syndrome in the course of internal malignancy.

The most common are hematologic malignancies apart from Sezary syndrome or mycosis fungoides. Also in publication we cannot find examples of carcinoma of the lung, [62] thyroid and prostate, adenocarcinoma of liver, ovarian, rectal and mammary cancer, malignant melanoma or esophageal carcinoma [63].

Typical symptoms of erythroderma include inflammation covering nearly 90% of the body, which may be accompanied by desquamation, excoriations and lichenification. Prolonged and severe erythroderma is often linked to diffuse alopecia, ectropion, nail deformities and keratoderma [64]. Other clinical features that can be observed involved pyrexia, general malaise, and pruritus [65]. Patients with erythroderma may experience tachycardia and high-output heart failure. Lymphadenopathy is typically related to this skin conditions and has been observed in the majority of erythrodermic patients. When lymphadenopathy occurs alongside organ enlargement, it may indicate drug hypersensitivity or malignancy [66].

Establishing a definitive clinicopathologic correlation may require several skin biopsies taken repeatedly. In extensive studies of samples from erythrodermic patients, the histopathological findings are frequently nonspecific, typically displaying hyperkeratosis/parakeratosis, acanthosis, and chronic inflammatory infiltrates (with or without eosinophils). Even in cases with established dermatoses, the biopsies can still yield non-diagnostic results. Although there are features that are often typical for cutaneous T-cell lymphoma (CTCL) like Pautrier’s microabscesses bandlike infiltrate with cerebriform nuclei and epidermotropism, multiple biopsies may be necessary to clearly identify these characteristics. Sézary syndrome might show minimal epidermotropism, necessitating additional clinical information for diagnosis [67]. Other conditions, like drug-induced pseudolymphoma, can mimic CTCL histology, complicating differential diagnosis with lymphadenopathy and hepatosplenomegaly. Several cases of Hodgkin’s lymphoma associated with erythroderma have revealed Reed–Sternberg cells in skin biopsies, whereas certain leukemias exhibit deep monomorphous infiltrates along with mixed superficial infiltrates. Generally, erythroderma linked to malignancy presents nonspecific histology. To distinguish malignant from benign infiltrates, techniques like immunophenotyping, flow cytometry, and ultrastructural morphometric analysis have been assessed, though they are not completely reliable due to overlaps [68].

Another case where erythroderma evolved into erythema gyratum repens, an obligate paraneoplastic dermatosis, is reported in the literature [69]. Paraneoplastic erythroderma can sometimes be distinguished from other forms of erythroderma by specific clinical features unique to the latter. If the condition is related to psoriasis, a family or personal history of psoriasis may indicate this background; additionally, typical nail changes (such as pitting, subungual keratosis, or oil spots) can also point toward this diagnosis [70]. If there are islands of healthy skin and the patient’s skin has a salmon hue, one should consider PRP [71]. Massive lymphadenopathy, ectropion, extensive skin infiltration, and recurrent erythroderma may suggest mycosis fungoides or Sézary syndrome [72,73]. The rapid onset of lesions and a history confirming the introduction of new medications, such as amoxicillin, allopurinol, carbamazepine, pregabalin, and nifedipine, may suggest a drug-induced background for the changes [74,75,76]. Atopic history, a young patient age, and pre-existing lesions typical of atopic dermatitis may suggest an exacerbation caused by this condition.

Identifying a cause requires correlation between patient history, clinical presentation, biopsy and direct immune fluorescent test findings, and laboratory studies. The differential diagnosis is presented in Table 1.

Malignancy-associated erythroderma may be more progressive and its course dictated by the prognosis of the underlying malignancy and response to therapy, while idiopathic erythrodermic cases may be unpredictable with periods of relapse and remission.

The initial treatment for all forms of erythroderma is consistent, irrespective of the underlying cause. Focus is placed on ensuring proper nutrition and replacing fluids and electrolytes. Specifically, any potential drugs inducing erythroderma must be stopped. Treatment includes antihistamines to reduce itching, emollients, and medium-potency topical steroids. In severe cases, systemic medications are necessary, such as intramuscular or oral corticosteroids and immunosuppressive drugs, especially when the changes are due to chronic conditions like psoriasis or atopic dermatitis. However, it is important to consider the potential underlying cause of erythroderma as an internal malignancy, as the use of immunosuppressants may worsen the underlying condition. Typically, proper treatment of the cancers leads to an improvement in the erythroderma as well [77].

## 3. Paraneoplastic Syndromes That Are Easier to Recognize

Paraneoplastic pemphigus (PNP) is a distinct form of pemphigus characterized by clinical, histological, and immunological features that are pathognomonic. As the name suggests, it is usually associated with an internal neoplasm. Two-thirds of cases present with a known malignancy at the time the eruption begins [78].

Recently, researchers have identified increased susceptibility to paraneoplastic pemphigus development in patients with HLA Class II Drb1*03 allele and HLA-Cw*14. These alleles are more common in Caucasian and Chinese populations, respectively. In contrast, HLA-DR4 and HLA-DR1-14 increase susceptibility to pemphigus vulgaris and pemphigus foliaceus, but have not shown an association with the development of PNP [79,80].

PNP is most frequently linked to B-cell lymphoproliferative disorders [81]. Another most common malignancies associated with PNP include chronic lymphocytic leukemia, Hodgkin’s lymphoma thymomas, Waldenström’s macroglobulinemia, sarcomas and Castleman’s disease [82].

**Figure 4:**
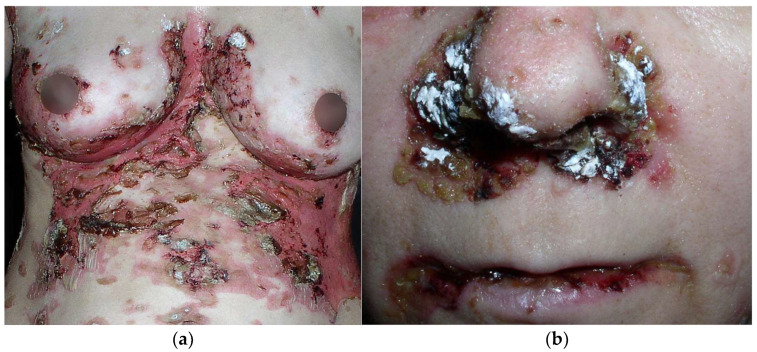
Paraneoplastic pemphigus. (**a**) Polymorphous skin lesions scattered on the trunk, including flaccid blisters and confluent erosions. (**b**) Lesions in the form of erosions covered with hemorrhagic and honey-yellow crusts, located on the skin and mucous membrane of the nasal cavity and lips.

PNP is characterized by polymorphous lesions that affect the skin and various mucous membranes. In almost all cases of PNP, oral lesions are seen, and are usually the first sign of the disease [83]. Mucosal lesions appear as widespread erosions and can involve the entire oral cavity, throat, or esophagus. Erosions in the lips are also frequently observed, where may be covered with crusts (Figure 4b). It is also important to consider changes in the anogenital areas. Cutaneous lesions typically appear after the onset of mucosal lesions [84]. Skin lesions usually spare the face. They may present as tense or flaccid blisters with erosions and can coexist with lichenoid or psoriasis-like eruptions. (Figure 4a). Ocular manifestation in PNP is also not uncommon. Bilateral conjunctival erosions may occur, which can lead to pseudomembranous conjunctivitis, symblepharon, and even vision loss [85]. In contrast to other forms of pemphigus, which primarily affect squamous epithelium, PNP can involve other types of epithelium, including those of the gastrointestinal and respiratory tracts. They can manifest as obstructive lung disease, bronchiolitis obliterans and may even result in respiratory failure [86].

To diagnose paraneoplastic pemphigus (PNP), Helm and Camissa established major and minor criteria. The main criteria include a polymorphous skin lesions, the presence of an associated internal malignancy, and the detection of antibodies through a specific immunoprecipitation standard. The additional criteria consist of intraepithelial acantholysis in histopathological examination, a linear pattern with IgG and C3 deposits along the basement membrane zone in direct immunofluorescence (DIF) examination and IIF using rat bladder epithelium as the substrate. For the diagnosis to be confirmed, either three major criteria or two major and one minor criterion must be met [82].

**Figure 5:**
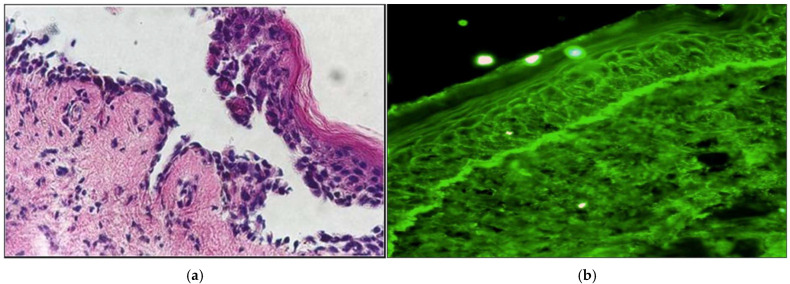
Paraneoplastic pemphigus. (**a**) Histopathological examination describes acantholysis in the lower epidermal layers, as well as hydropic degeneration of the basal layer and necrosis of individual keratinocytes. (**b**) IgG and C3 deposits arranged in a reticular pattern and linearly along the basement membrane.

Histopathological changes depend on the clinical features of the lesions. The most commonly observed characteristics include suprabasal acantholysis, dyskeratotic keratinocytes, which may be present in all layers of the epidermis, a dense lichenoid infiltrate at the dermal–epidermal junction and migration of inflammatory cells into the epidermis [87]. The histopathological examination of paraneoplastic pemphigus are presented in Figure 5a.

Direct immunofluorescence (DIF) on a perilesional biopsy can show intercellular IgG and C3 deposits. Additionally, linear IgG or C3 deposits at the basement membrane zone, caused by autoantibody binding to BPAG1, help distinguish PNP from other pemphigus types, where Ig deposits are limited to keratinocytes [88]. A positive DIF result is not required to confirm a diagnosis of PNP (approximately 50% cases of PNP have negative DIF). Also false-negative results are often, especially from oral mucosa, because of a lot of necrotic tissue. The characteristic direct immunofluorescence pattern is shown in Figure 5b.

Indirect immunofluorescence using rat bladder is a key diagnostic tool for PNP, as the presence of autoantibodies to plakins is a hallmark feature. The most specific markers are autoantibodies against envoplakin and periplakin, followed by desmoplakin I and II. Immunoblotting and ELISA are alternative methods for detecting plakin autoantibodies in PNP. Immunoblotting can identify antibodies against desmoplakin I and II, periplakin, and envoplakin using cultured human keratinocyte extracts. Additionally, recombinant fragments of periplakin and envoplakin can also be utilized in both immunoblotting and ELISA for detection [89]. Proteins targeted by autoantibodies in paraneoplastic pemphigus are presented in Table 2 [84,90].

PNP can present a diagnostic challenge, especially when the skin lesions resemble other conditions. In differential diagnosis, it is important to consider other blistering diseases, including pemphigus vulgaris, pemphigus foliaceus, pemphigoid, as well as erythema multiforme, toxic epidermal necrolysis, systemic lupus erythematosus, graft-versus-host disease and lichen planus [91,92,93]. If extensive erosive lesions, including mucosal involvement, predominate, they may be mistaken for erythema multiforme or toxic epidermal necrolysis.

In such cases, a patient history indicating the use of a new medication can aid in differentiation. Additionally, direct and indirect immunofluorescence testing in PNP will confirm the presence of characteristic immunoglobulin deposits. Tense blisters, particularly on the lower legs, accompanied by mucosal erosions, may suggest bullous pemphigoid. In such cases, the patient’s age, associated conditions and symptoms, and the definitive role of direct immunofluorescence (DIF) testing are crucial for differentiation [94]. Targetoid and lichenoid lesions may also occur over the palms and soles in addition to blisters. When the lesions are papular, they may resemble lichen planus. Especially, after treatment and in chronic cases, the eruption is predominantly lichenoid in nature. In such cases, a medical history indicating recurrent lichen planus-like lesions can provide a clue for diagnosis. Histopathological examination of a biopsy taken from the lichenoid lesion area may be inconclusive. In these situations, the final diagnosis is established based on immunofluorescence studies [95]. In cases of severe mucosal lesions, especially within the oral cavity, systemic lupus erythematosus (SLE) should also be considered in the differential diagnosis. This disease can also present with ulcers in this region. Additionally, there is a bullous form of SLE, characterized by blisters with erosions on the skin. In such cases, antinuclear antibody (ANA) testing may be helpful, as it often points toward an SLE diagnosis (e.g., anti-dsDNA or Sm antibodies, which are typical for SLE). DIF may not always be a definitive test in this scenario due to possible positive findings of IgG, IgM, IgA, or C3 deposits along the dermoepidermal junction, known as the lupus band test. Rare cases of coexistence of both diseases have also been described in the literature [96,97]. When erosions and flaccid blisters predominate, PNP can be mistaken for pemphigus vulgaris or pemphigus foliaceus. In such cases, both DIF and IIF should be helpful in establishing the correct diagnosis [98]. For patients with paraneoplastic pemphigus (PNP) and no apparent malignancy, the diagnostic evaluation should be specifically directed towards identifying potential underlying cancers. The management of paraneoplastic pemphigus (PNP) is dual focused. On the one hand, treatment should target the associated malignancy; on the other hand, therapy must address the lesions caused by PNP [99]. Prognosis tends to be better in cases of less aggressive malignancies, such as thymoma or Castleman’s disease. Initial treatment typically involves high doses of systemic steroids. However, due to the adverse effects associated with long-term use of high-dose steroids, additional immunosuppressive agents, such as cyclosporine, azathioprine, or cyclophosphamide, should be included. In some cases, intravenous immunoglobulin infusions or plasmapheresis may be employed. In recent years, an increasing number of blistering disease cases have been treated with rituximab (RTX), a chimeric human-murine anti-CD20 monoclonal antibody. New studies have emerged confirming the effectiveness of RTX also in paraneoplastic pemphigus [100]. Cases have also been described in the literature where the treatment of the underlying disease, such as chronic lymphocytic leukemia with alemtuzumab, led to complete remission of lesions in coexisting paraneoplastic pemphigus [101,102]. Despite available treatments, PNP remains associated with high mortality [103]. A significant risk of death is primarily associated with respiratory failure due to bronchiolitis obliterans. This results from the accumulation of antibodies not only in the skin but also in the respiratory tract, leading to progressive airway narrowing, breathing difficulties, and ultimately death [104]. According to some authors, this cause accounts for more than 90% of all deaths in the course of PNP [105]. It is also important to consider the adverse effects of immunosuppressive therapy, increased susceptibility to infections, and the potential progression of the underlying malignancy, which triggered PNP, as potential causes of patient mortality.

Dermatomyositis (DM) is an infrequent autoimmune disease with numerous cutaneous and systemic symptoms. Dermatomyositis primarily manifests with skeletal muscle weakness, multiple skin abnormalities and extramuscular symptoms such as esophageal motility disorder (EMD) and interstitial lung disease (ILD) [106].

Although the exact cause of this condition remains unclear several risk factors as genetic predisposition, exposure to UV radiation, geographic and environmental factors are believed to play a major role in its development.

One of the risk factors is the presence of a specific type of human leukocyte antigen (HLA). Studies have confirmed a correlation between the haplotypes HLA-A*68 in North American Whites, HLA-DRB1*0301 in African Americans, HLA-DQA1*0104 and HLA-DRB1*07 in Han Chinese, DQA1*05 and DQB1*02 in Britons and an increased risk of developing dermatomyositis, as well as the haplotype DRB1*03-DQA1*05-DQB1*02 and the risk of developing interstitial lung disease in dermatomyositis [107,108].

The predominant age for DM is considered to be between 40 and 50 years old, and it occurs twice as often in women than in men [109]. The chronic nature of dermatomyositis is commonly characterized by periods of clinical remissions and exacerbations, which may also occur without noticeable changes in muscle function (amyopathic dermatomyositis).

**Figure 6:**
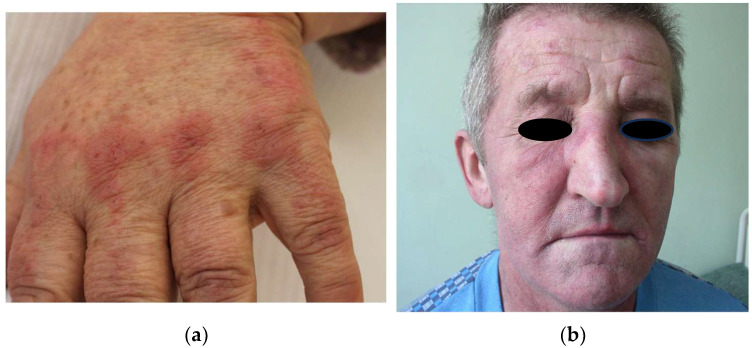
Pathognomic signs of dermatomyositis. (**a**) Erythematous papules located over the metacarpophalangeal joints. (**b**) Violaceous rash affecting upper eyelids with periorbital edema.

Diversified skin abnormalities can be present in the course of this disorder. Literature data distinguish pathognomonic symptoms as Gottron papules which are characterized by the presence of violaceous papules above the metacarpophalangeal and interphalangeal joints on hands, as shown in Figure 6a. Other findings consist of heliotrope rash (red or pink erythema of the eyelids accompanied by swelling of affected tissue—presented in Figure 6b), purple erythema on dorsal surfaces of the hands, elbows, knees and ankles (Gottron’s symptom), V-neck erythema and erythema of the neck and shoulders (scarf symptom), calcium deposits in the skin, etc. In many cases, cutaneous manifestations can be accompanied by photosensitivity and itching [110]. Symptoms of dermatomyositis are presented in Table 3.

In the diagnosis of DM, in addition to typical clinical symptoms, supportive tests also play a significant role. Elevated levels of muscle enzymes are observed in 95% of patients, especially aldolase and creatine kinase. Less specific enzymes, such as aspartate aminotransferase, alanine aminotransferase, and lactate dehydrogenase, may also be elevated [111]. Another diagnostic tool for detecting muscle inflammation in DM is electromyography (EMG). Electromyography is typically conducted on a proximal muscle, like the triceps. A positive EMG result largely confirms the presence of muscle inflammation in the course of DM [112]. Due to its non-invasive nature, MRI of the muscles is also gaining popularity and can be helpful in diagnosing muscle inflammation [113].

However, a skin biopsy is not always diagnostic and may show findings similar to the histopathology of systemic lupus erythematosus (vacuolar changes in the basal layer, increased lymphocytic infiltrate, and increased mucin deposition in the dermis), muscle biopsy often shortens the diagnostic process and confirms diagnosis [114].

Typical abnormalities observed in muscle histopathology include perivascular and perimyosial inflammatory infiltrate (associated with the presence of B lymphocytes, CD4+ helper T lymphocytes, macrophages, and plasmacytoid dendritic cells, which distinguishes the disease from polymyositis, where CD8+ lymphocytes and NK cells predominate), and perifascicular atrophy affects the edges of muscle fascicles and microangiopathy, which is caused by the deposition of immunoglobulins and the complement complex (C5b-C9) [110,115].

Various studies have shown that dermatomyositis has a strong correlation with malignancies [116,117]. Over 30% of adult patients with DM are at risk of developing cancer and it can be linked with the existence of specific autoantibodies. Although certain autoimmune antibodies are thought to contribute to the development of myositis in cancer patients, the exact mechanism remains unclear. Studies suggest that transcription mediator factor 1-gamma (TIF1-γ), released by cancer cells, triggers the production of TIF1-γ antibodies, which in turn can lead to the development of dermatomyositis [118].

Many studies confirmed that patients with detectible anti-TIF1-γ (transcription intermediary factor, anti-p155) and TIF1-α (anti p-140) antibodies were further diagnosed with cancer or other malignancies [119]. According to the research conducted by Yumi Harada’s team, among 14 patients with dermatomyositis (DM) who tested positive for anti-TIF1γ antibodies, 86% were found to have an associated malignancy. This confirms the strong correlation between DM with TIF1γ antibodies and its role as a paraneoplastic syndrome [120]. Immunological assessment of each DM patient can be a useful tool to forecast malignancy risk in the course of the disease. All adults confirmed with DM should undergo full screening to rule out possibility of malignant process. DM can occur before, concurrently or after diagnosis of cancer.

Malignancies related to DM include ovary, lung, pancreas, breast and gastrointestinal tract cancers. Non-Hodgkin’s lymphoma, testicular cancer and nasopharyngeal carcinoma were also described [121]. The most common malignancy associated with dermatomyositis is ovarian cancer [122]. The goal of the treatment is to obtain improvement in patient comfort and daily activities, reduce muscle weakness and avoid systemic complications (including ILD, arthritis, and cutaneous lesions).

Considering initial therapy, systemic corticosteroids are still believed to be the finest option yet still less effective in patients with underlying malignancy. Other considered options consist of intravenous immunoglobulin (IVIG), rituximab, methotrexate, and hydroxycholoroquine. Research into new therapeutic options for patients with dermatomyositis is ongoing, and new biologic drugs under investigation include efgartigimod, nipocalimab, dazukibart, brepocitinib, and lenabasum [123]. Conclusively, the optimal and most favorable therapy in DM patients with concomitant malignancies is to coordinate oncological treatment along with immunosuppressants [124].

Leser–Trélat syndrome is a rare paraneoplastic sign in which uncontrollable eruption of multiple seborrheic keratoses is associated with underlying malignancy. So far, no diagnostic criteria have been estimated and disease is confirmed on clinical data itself.

The Leser–Trélat sign is oftentimes related to breast and gastrointestinal adenocarcinomas but renal, hepatic, and pancreatic malignancies were also reported in literature data. The definite cause of this phenomenon is not fully elucidated; however, it is suspected that several growth factors (especially epidermal growth factor (EGFR)) secreted from the tumor stimulate the uncontrolled growth of seborrheic keratosis [125].

The precise pathophysiology of the Leser–Trélat sign remains unclear. It is thought that the phenomenon may result from cytokines and growth factors released by the neoplasm, which stimulate the rapid proliferation of seborrheic keratoses. The main growth factors include epidermal growth factor-α (EGF-α), transforming growth factor-α (TGF-α), and amphiregulin, as well as human growth hormone, fibroblast growth factor receptor 3 (FGFR3), phosphatidylinositol 3-kinase catalytic subunit alpha (PIK3CA), and insulin-like growth factor [2,126,127].

Seborrheic keratoses are benign epidermal neoplasms arising from clonal immature keratinocytes and have tendency to appear frequently, especially in in patients aged >40 years. The characteristics and frequency of those lesions induce skepticism regarding the actual occurrence of this disease. Multiple seborrheic keratosis have been reported not only in cancerous patients, but also in the course of infectious diseases, such as HIV, COVID-19 infection or in post-transplant patients. The cause of this phenomenon is not fully understood [128]. The morphology of these benign neoplasm is manifested by the presence of well-demarcated macules, papules, and plaques with a variety of colors. Pigmentation ranges from light to dark brown or black. In consideration of color, they have a tendency to vary extensively amongst patients. The surface has a soft, greasy or scaly consistency, with diversity of size. Diagnosing seborrheic keratosis is usually straightforward. Dermoscopic examination reveals common findings as comedo-like (CL) openings, fissures and ridges (FR), and milia-like (ML) cysts [129].

In doubtful or uncertain cases, histopathology is the procedure of choice. Regardless of the genesis of these lesions, histopathological examination presents identical features [130]. Literature data estimated that the most prevalent histopathological type of seborrheic keratosis is acanthotic type, followed by mixed, hyperkeratotic, melanoacanthoma, clonal, irritated and adenoid types. Histological assessment is performed to rule out other skin lesions, including skin cancers.

Roh NK et al. proved that other pathologies that were mistaken for SK included the common wart, flat wart, basal cell carcinoma, squamous cell carcinoma, dysplastic nevus, compound nevus, actinic keratosis, Bowen disease, melanoma and condyloma acuminatum [131].

The tumors associated with Leser–Trélat syndrome are presented in Table 4 [132,133,134,135].

The therapy of concomitant cancer is of fundamental importance in the treatment of this disease, Leser–Trélat syndrome. It is declared that approximately 50% of associated seborrheic keratosis resolve completely. Additionally, patient disturbing lesions may be managed with cryotherapy, curettage and electrodessication [136].

Acanthosis nigricans (AN) is a symptom that can be classified as benign or malignant but then is associated with malignant tumors. Acanthosis nigricans appears as hyperpigmented skin with excessive keratinization, most commonly symmetrically affecting the armpits, groin, and the skin on the back of the neck. Acanthosis nigricans can also be localized on the face, oral mucosa and anogenital areas. Additionally, there could be hyperkeratosis of palms and soles, knuckles and fingers.

The distribution of lesions is often related to the underlying cause of AN. For example, facial lesions are most commonly observed in cases of metabolic syndrome, while involvement of the eyelids and mucous membranes may be a marker of malignancy [137,138]. Patients often complain of itching in the areas of skin lesions.

Several factors have been described as playing a role in the development of acanthosis nigricans. One of these may be an increased level of insulin in the blood, which can displace insulin-like growth factor 1 (IGF-1) from its binding proteins, consequently leading to an increase in its concentration and excessive proliferation of keratinocytes and dermal fibroblasts. The changes can also occur as a hereditary variant due to a genetic defect in fibroblast growth factor (FGF), which is important for cell growth, repair, and angiogenesis. In rare cases, the changes are associated with the presence of tumors. In such cases, there is an increased amount of transforming growth factor (TGF), leading to abnormal skin growth through the epidermal growth factor receptor [139].

Mild forms of AN are most often associated with endocrine disorders, particularly those linked to metabolic syndrome and insulin resistance. It is also common for patients with diabetes to develop lesions typical of AN [140]. Moreover, it can be triggered by specific medications, such as corticosteroids, nicotinic acid, and triazinate [20]. The link between acanthosis nigricans and internal malignancy was initially identified by Darier in 1893 [141].

“Malignant” acanthosis nigricans (MAN) is primarily diagnosed in individuals over the age of 40. The most commonly associated cancer is gastrointestinal adenocarcinoma. There have been reported cases where AN was found alongside tripe palms, mucosal papillomas, and the Leser–Trélat sign, especially when associated with gastric cancer [142,143]. Less commonly, AN may be a syndrome associated with carcinomas of the lung, kidney, and bladder, as well as mycosis fungoides and carcinomas of the ovary and pancreas [144].

The correct diagnosis can be assisted by a histopathological analysis of the skin lesions. The features of acanthosis nigricans are distinctive, indeed pathognomonic, and typically involve significant papillomatosis with “finger-like” projections of the rete ridges. Acanthosis is generally mild and rather restricted to the valleys between the papillomatous formations. Hyperkeratosis is also observed, but it resembles a basket-weave pattern [145]. Although the changes in acanthosis nigricans (AN) are quite characteristic clinically, they can sometimes be mistaken for other conditions, especially in the early stages.

Conditions like hypothyroidism, Cushing’s syndrome, and polycystic ovary syndrome (PCOS) may also present with hyperpigmentation similar to AN. In such cases, hormonal and imaging tests are decisive. Chronic irritation or friction, especially in skin folds, can also lead to hyperpigmentation that might mimic AN. Due to their location in skin folds, the changes may initially be mistaken for fungal intertrigo. Under these circumstances, mycological examination can be helpful for differentiation.

After diagnosing acanthosis nigricans, it is essential to explore possible underlying causes, especially if skin lesions appear in adulthood for the first time. When factors like family history, endocrine disorders, obesity or associated medications are not present, the emergence of this condition should prompt an evaluation for potential malignancy, with a focus on the gastrointestinal tract [146]. Once the tumor is removed, the condition may regress. This suggests that growth factors produced by the tumors could play a causal role in the development of AN.

The treatment of acanthosis nigricans focuses on regulating keratinocyte proliferation. Local preparations containing retinoids (e.g., tretinoin or mix of ammonium lactate with tretinoin) [147,148], podophyllin, and vitamin D analogs are used for this purpose. Systemic keratolytics may be applied, and in the case of a metabolic underlying cause, such as insulin resistance, metformin may be used [149].

## 4. Conclusions

This article has explored the various disease entities within the realm of paraneoplastic skin syndromes (PNS), highlighting their critical role as potential early indicators of underlying malignancies. The discussion emphasized the necessity for healthcare professionals, including dermatologists, oncologists, and primary care physicians, to maintain a high index of suspicion for PNS. Recognizing and differentiating these syndromes from other dermatological conditions are essential, despite the inherent challenges posed by their rarity and diverse clinical presentations. The summary of the most important information regarding the discussed paraneoplastic syndromes is presented in Table 5. This article underscores the importance of continuous education and dialogue among medical practitioners to improve early detection and patient outcomes. By fostering awareness and knowledge sharing, the medical community can better navigate the complexities of PNS and enhance interdisciplinary collaboration in the diagnosis and management of associated malignancies.

## Figures and Tables

**Table 1:** Differential Diagnosis of Erythroderma.

Category	Condition
Metabolic and Endocrine Disorders	Acanthosis nigricans
Granulomatous Diseases	Acute complications of sarcoidosis
Malignancies	Cutaneous T-cell lymphoma
Autoimmune Blistering Diseases	Bullous pemphigoid, pemphigus foliaceus
Allergic Diseases	Irritant contact dermatitis, pediatric atopic dermatitis, allergic contact dermatitis
Inflammatory Skin Diseases	Plaque psoriasis, lichen planus, pityriasis rubra pilaris, seborrheic dermatitis, stasis dermatitis
Rheumatologic Diseases	Reactive arthritis
Genodermatosis	Familial benign pemphigus (Hailey–Hailey disease)
Other Diseases	Dermatologic manifestations of graft-versus-host disease

**Table 2:** Proteins targeted by autoantibodies in PNP.

Protein Name	Molecular Weight (kD)	Location
Desmoglein type 1 (Dsg1) Desmoglein type 3 (Dsg3)	160 130	Desmosome, extracellular
Desmoplakin 1 Desmoplakin 2	250 210	Desmosome, intracellular
Bullous pemphigoid antigen 1 (BP230)	230	Hemidesmosome/lamina lucida
Envoplakin	210	Desmosome, intracellular
Epiplakin	>700	Desmosome, intracellular
Periplakin	190	Desmosome, intracellular
Alpha-2-macroglobulin-like-1-antigen (A2ML1)	170	Protease inhibitor

**Table 3:** The symptoms of dermatomyositis.

	Skin Finding	Description
Pathognomic symptoms	Gottron papules	red or purple papules, with or without scaling or ulceration located over the interphalangeal or metacarpophalangeal joints.
Other symptoms	Heliotrope rash	red or pink erythema of the eyelids accompanied by swelling of affected tissue; may be less noticeable in patients with dark skin
	Gottron symptom	purple erythema on dorsal surfaces of the hands, elbows, knees and ankles
	Facial erythema	redness across the cheeks and nose, involving the nasolabial folds, sometimes extending to the forehead and ears
	Scarf symptom	redness on the back of the neck, upper back, and shoulders, sometimes extending to the upper arms
	V sign	ill-defined red patches on the front of the neck and upper chest
	Calcinosis cutis	calcium deposits in the skin

**Table 4:** The tumors associated with Leser–Trélat syndrome.

The Most Common Causes	The Rarer Causes
Adenocarcinomas - gastrointestinal adenocarcinomas are commonly associated - gastric adenocarcinoma is the most common	Breast cancer Hematopoietic neoplasms Lung cancer Pancreatic malignancy Prostate malignancy Kidney malignancy Laryngeal malignancy Ovarian and uterine malignancy Bladder malignancy Nasopharyngeal carcinoma Melanoma Mycosis fungoides Hepatocellular carcinoma Squamous cell carcinoma

**Table 5:** Summary of the most important information regarding the discussed paraneoplastic syndromes.

Paraneoplastic Syndromes	The Most Common Causes	Clinical Presentation	Differential Diagnosis	Additional Tests	Treatment Options
Erythema gyratum repens	cancers: lung, breast, esophageal, cervical, stomach, pharyngeal others: bullous pemphigoid, CREST syndrome, tuberculosis, PRP	rapid spreading erythema “zebra skin” lokalization: trunk, proximal extermities pruritus	tinea corporis, psoriasis, DLE, bullous pemhigoid, erythema migrans, erythema annulare centrifugum	histopathology: hyperkeratosis, parakeratosis, spongiosis, lymphohistiocytic infiltrate around dermal vessels, eosinophils, mast cells DIF: granular IgG and C3 deposition along the dermal–epidermal junction	cancer treatment is crucial corticosteroids and azathioprine are often ineffective
Necrolytic migratory erythema	cancers: glucagonoma—the most common, lung others: zinc deficiency, liver disease, pancreatitis	reddish-brown plaques undergoing superficial necrosis and crusting localization: perioral region, trunk, extermities, perineum pruritus, pain, burning	acrodermatitis enteropathica, erythema multiforme, contact or atopic dermatitis, erythrokeratoderma, psoriasis, Hailey–Hailey disease	histopathology: necrosis of the upper spinous layer, irregular acanthosis, loss of granular layer, parakeratosis over vacuolated keratinocytes, perivascular lymphocytic or neutrophilic infiltrate, intraepidermal bullae	cancer treatment is crucial somatostatin analogs, INF-alpha, biological agents: sunitinib, everolimus, cryoblation
Bazex syndrome	cancers: lung, oral, hand and neck, esophageal	scaly, hyperkeratotic, red to purple eruptions localization: hands, feet, ears, nose	psoriasis, fungal infection, eczema	histopathology: parakeratosis, hyperkeratosis, isolated necrosis of keratinocytes, acanthosis, perivascular lymphohistiocytic inflammatory infiltrate DIF: usually negative results	cancer treatment is crucial systemic and topical retinoids, systemic and topical steroids, PUVA therapy, zinc supplementation
Acute febrile neutrophilic dermatitis	leukemia, embryonal carcinoma of testis, ovarian and gastric carcinoma, adenocarcinoma of breast, prostate, and rectum others: infections, e.g., gastrointestinal, tuberculosis, HIV; autoimmune conditions, e.g., rheumatoid arthritis; drug-induced; idiophatic; pregnancy	red, painful, well-demarcated plaques or nodules; typically pseudovesicular/ pseudopustular localization: face, neck, back, chest, extermities arthritis, pyrexia	erythema nodosum, toxic pustuloderma, periarteritis nodosa, granuloma faciale, leukocytoclastic vasculitis, erythema elevatum diutinum, erysipelas, impetigo contagiosum	histopathology: widespread neutrophilic infiltrate in the dermis, with fragmentation and edema of neutrophil nuclei, sometimes spongiotis vesicles or subcorneal pustules	cancer treatment is crucial systemic and topical steroids, anti-inflammatory/immunosuppressive agents, potassium iodide, colchicine
Pyoderma gangrenosum	myeloproliferative neoplasms, breast, colon, bladder, prostate cancers others: arthritis, inflammatory bowel disease, and paraproteinemia	an irregular, painful ulcer developing from an initial single lesion, such as a pustule; erythema in the surrounding area “sieve sign” and “pathergy phenomenon” localization: lower limbs, trunk	venous or arterial insufficiency, Sweet syndrome, antiphospholipid syndrome, systemic lupus erythematosus, venous or arterial ulcers, tuberculosis, sporotrichosis, ulcerating skin tumors, lymphomas, vasculitis	histopathology: samples from the ulcer’s edge-neutrophils and perivascular lymphocytic infiltrates along with dermal edema samples from the center of the ulcer- neutrophilic infiltrate; vascular damage	cancer treatment is crucial systemic steroids, immunosuppressive drugs, dapsone, colchicine, TNF-alpha inhibitors careful surgical wound debridement
Exfoliative dermatitis	hematologic malignancies—Sezary syndrom, mycosis fungoides carcinoma of the lung, thyroid, prostate, adenocarcinoma of liver, ovarian, rectal and mammary cancer, malignant melanoma, esophageal carcinoma others: exacerbation of pre-existing dermatological conditions	inflammation covering nearly 90% of the body desquamation, excoriations and lichenification pyrexia, general malaise, pruritus, lymphadenopathy	acanthosis nigricans, acacute complications of sarcoidosis, cutaneous T-cell lymphoma, autoimmune blistering diseases, allergic diseases, inflammatory skin diseases, reactive arthritis, familial benign pemphigus, graft-versus-host disease	histopathology: hyperkeratosis, parakeratosis, acanthosis,chronic inflammatory infiltrates	cancer treatment is crucial antihistamines, topical and systemic steroids, immunosuppressive drugs
Paraneoplastic pemphigus	B-cell lymphoproliferative disorder, chronic lymphocytic leukemia, Hodgkin’s lymphoma thymomas, Waldenström’s macroglobulinemia, sarcomas, Castleman’s disease	polymorphic lesions—erosions, blisters, lesions resembling erythema multiforme, lichen planus, etc. usually initially on the mucous membranes, followed by the skin, may involve the mucous membranes of internal organs	pemphigus vulgaris, pemphigus foliaceus, pemphigoid, erythema multiforme, toxic epidermal necrolysis, systemic lupus erythematosus, graft-versus-host, lichen planus	histopathology: suprabasal acantholysis, dyskeratotic keratinocytes, a dense lichenoid infiltrate at the dermal–epidermal junction, migration of inflammatory cells into the epidermis DIF: intercellular IgG and C3 deposits, linear IgG or C3 deposits at the basement membrane zone IF, Immunoblotting and ELISA: autoantibodies against envoplakin and periplakin, followed by desmoplakin I and II	cancer treatment is crucial high-dose steroids, additional immunosuppressive agents, intravenous immunoglobulin infusions, plasmapheresis, rituximab
Dermatomyositis	ovary, lung, pancreas, breast, gastrointestinal tract, testicular cancers, Non-Hodgkin’s lymphoma, nasopharyngeal carcinoma	Gottron papules, heliotrope rash, Gottron’s symptom, V-neck erythema, scarf symptom, calcium deposits in the skin photosensitivity, itching skeletal muscle weakness, esophageal motility disorder, interstitial lung disease	systemic lupus erythematosus	elevated levels of muscle enzymes the features of muscle inflammation on EMG and MRI histopathology: -skin biopsy: vacuolar changes in the basal layer, increased lymphocytic infiltrate, increased mucin deposition in the dermis -muscle biopsy: perivascular and perimyosial inflammatory infiltrate, perifascicular atrophy, microangiopathy	cancer treatment is crucial systemic corticosteroids, intravenous immunoglobulin (IVIG), rituximab, methotrexate, hydroxycholoroquine, new biologic drugs under investigation
Leser–Trélat syndrome	breast, gastrointestinal adenocarcinomas, renal, hepatic, and pancreatic malignancies others: infectious diseases—HIV, COVID-19, post-transplant patients	well-demarcated macules, papules and plaques with soft, greasy, or scaly consistency, pigmetation ranges from light brown to dark brown	common wart, flat wart, basal cell carcinoma, squamous cell carcinoma, dysplastic nevus, compound nevus, actinic keratosis, Bowen disease, melanoma, condyloma acuminatum	dermoscopic examination: comedo-like openings, fissures and ridges, milia-like cysts the most prevalent histopathological type is acanthotic type	cancer treatment is crucial cryotherapy, curettage, electrodessication
Acanthosis nigricans	gastrointestinal adenocarcinoma, carcinomas of the lung, kidney, ovary, pancreas and bladder, mycosis fungoides others: metabolic syndrome, insulin resistance, drug induced	hyperpigmented skin with excessive keratinization localization: armpits, groin, and back of the neck; itching	hypothyroidism, Cushing’s syndrome, and polycystic ovary syndrome, chronic irritation or friction, especially in skin folds, fungal intertrigo	histopathology: significant papillomatosis with “finger-like” projections of the rete ridges, hyperkeratosis, resembling a basket-weave pattern, acanthosis restricted to the valleys between the papillomatous formations	cancer treatment is crucial retinoids, podophyllin, vitamin D analogs, metformin
